# Self-Management at Work’s Moderating Effect on the Relations Between Psychosocial Work Factors and Well-Being

**DOI:** 10.3390/ijerph22071070

**Published:** 2025-07-03

**Authors:** Carol-Anne Gauthier, Tyler Pacheco, Élisabeth Proteau, Émilie Auger, Simon Coulombe

**Affiliations:** 1 Champlain Regional College—St. Lawrence, 790 Nérée Tremblay, Québec, QC G1V 4K2, Canada; 2 Department of Industrial Relations, Université Laval, 2325 Rue de l’Université, Québec, QC G1V 0A6, Canada; 3 Relief Research Chair in Mental Health, Self-Management, and Work, Université Laval, 2325 Rue de l’Université, Québec, QC G1V 0A6, Canada; 4 VITAM—Sustainable Health Research Centre, 2480, Chemin de la Canardière, Québec, QC G1J 2G1, Canada; 5 Department of Psychology, Université de Sherbrooke, 2500, Boul. de l’Université, Sherbrooke, QC J1K 2R1, Canada; 6 Collège Ahuntsic, 9155, Rue St-Hubert, Montréal, QC H2M 1Y8, Canada; 7 CERVO Brain Research Centre, 2601 Chemin de la Canardière, Québec, QC G1J 2G3, Canada; 8 Centre d’Études et d’Interventions en Santé Mentale, Université Laval, 2325 Rue de l’Université, Québec, QC G1V 0A6, Canada; 9 Centre for the Study of Democratic Citizenship, Department of Political Science, Université de Montréal, C.P. 6128 Succursale Centre-Ville, Montréal, QC H3C 3J7, Canada

**Keywords:** self-management strategies, mental health self-management, self-care, workplace well-being, psychosocial work factors, positive mental health, psychological health

## Abstract

Mental health self-management (MHS) strategies may help workers with mental health concerns preserve and enhance their well-being. However, little research has explored how these strategies may help mitigate the effects of negative psychosocial work factors (PWFs) on well-being outcomes. This cross-sectional study investigated (1) the relationship between PWFs and well-being, (2) the association between MHS at work and well-being, and (3) the moderating role of self-management in preventing negative PWFs’ deleterious effects. A sample of 896 Francophone workers in Canada completed a questionnaire that included self-reported measures related to workplace, self-management, and well-being. Structural equation modeling (conducted via the MPlus software, version 8.6) revealed that psychological demands were negatively related to positive well-being outcomes and positively associated with adverse well-being outcomes. Competency-related autonomy was positively associated with flourishing, and recognition was positively associated with flourishing and positive well-being at work, as well as being negatively associated with burnout and depression. Surprisingly, supervisor support was negatively related to positive well-being and positively related to burnout and depression. MHS was positively associated with positive well-being at work, flourishing, and work performance, but had no relationship with negative mental health. MHS significantly moderated the relationship between each PWF and well-being at work in both beneficial and adverse ways, depending on the specific well-being indicator being considered. From a workplace well-being perspective, this suggests that although self-management may help workers preserve and enhance their positive well-being, organizations must also directly target PWFs to prevent negative well-being outcomes.

## 1. Introduction

Workplace well-being has garnered increased attention in recent years. It has been of the utmost importance for many employers since the COVID-19 pandemic, given the significant consequences it has had for both individuals and the organizations they work for [1], in areas such as job satisfaction, engagement, sickness absenteeism, productivity, employee turnover, and mental health-related disability insurance costs [2,3,4,5]. Central to addressing workplace well-being challenges is acknowledging and acting upon psychosocial work factors (PWFs), as they can have a significant influence on well-being.

### 1.1. Theoretical Models and Literature Review

#### 1.1.1. Psychosocial Work Factors and Well-Being

PWFs are work-related characteristics (e.g., psychological demands, decisional authority, social support) that can influence workers’ and organizations’ health and performance-related outcomes, such as burnout, engagement, and motivation [6,7,8,9,10,11]. They are often studied using the job demands and resources (JDR) model, which posits that high psychological demands (e.g., high workload, role ambiguity) and insufficient resources (e.g., low social support, lack of feedback) can lead to negative health outcomes, such as exhaustion and depression [6,7]. Decades of research have shown a clear relationship between “negative” PWFs (i.e., psychosocial risk factors) and adverse physical and mental health outcomes (e.g., increased cardiovascular disease and mental ill-being) [12]. A growing body of work also explores how “positive” PWFs such as job control, social support, job security, and stimulating, challenging, and meaningful work are associated with positive well-being, i.e., high subjective well-being and functioning [13,14,15,16,17].

Moreover, the relationships between PWFs and well-being can be moderated and mediated by personal resources (e.g., self-efficacy) and individual strategies (e.g., job crafting) [7,18]. Broadly defined, individual strategies refer to the coping mechanisms used to manage one’s energy levels, motivation, and overall functioning. These can include strategies aimed at changing one’s work environment (e.g., job crafting [19]) and influencing one’s psychological states and energy levels (e.g., proactive vitality management [20]). One such individual strategy might be mental health self-management at work, that is, the practices individuals use to preserve or enhance their mental health at work [21,22]. However, mental health self-management has rarely been studied in the context of work [22].

Mental health self-management at work (MHSW) refers to behavioral, cognitive, and affective strategies individuals use to manage and optimize their well-being [23,24] in relation to their work [22]. Although previous research has focused on the use of mental health self-management strategies in individuals with mental health concerns (e.g., depression [21]) and the effects of these strategies on mental health outcomes (e.g., depressive symptoms), the current study measured self-reported mental health self-management strategy use in the general working population, i.e., in individuals with and without diagnosed mental health concerns, as well as broader well-being outcomes such as flourishing.

#### 1.1.2. Mental Health Self-Management (MHS)

Self-management is the process in which an individual develops the responsibility, power, motivation, and will to act on improving their condition [23,25,26]. A key component of self-management is promoting empowerment, i.e., the degree of control an individual has over their environment and the perception of this control [27]. Empowerment is vital for self-management, as individuals who utilize this tool make numerous daily decisions regarding their health [23,25,26].

The concept of self-management was developed in the field of chronic diseases and medical rehabilitation to help patients manage chronic physical illnesses such as arthritis [28] and diabetes [29]. Self-management was then adapted to manage chronic mental health disorders such as schizophrenia, schizoaffective disorders, mood disorders, anxiety disorders, substance abuse, and others [30,31,32,33]. It is based on the same principles as the self-management of physical chronic illnesses and involves monitoring and managing one’s symptoms to prevent relapse and enhance one’s mental health [23,25,26]. This involves the use of daily strategies to regulate symptoms, modify lifestyle habits, prevent relapse, and improve overall health [23,34]. Practices that enhance one’s well-being can also relate to other components of one’s health, such as physical health [35]. Self-management does not replace traditional treatments (e.g., therapy, medication) but can be used in conjunction with them [23]. In addition, other factors, such as health literacy (i.e., “the degree to which individuals can obtain, process, understand, and communicate about health-related information needed to make informed health decisions” [36] (p. 16), influence individuals’ abilities to harness these tools towards recovery [37].

A series of randomized controlled trials have shown that mental health self-management programs provide many benefits to participants with mental health conditions, such as psychiatric symptom reduction and improvements in self-efficacy, hope, empowerment, quality of life, mental health, and overall health [30,33,38,39,40,41,42].

#### 1.1.3. Mental Health Self-Management at Work

Mental health self-management at work (MHSW) refers to strategies that individuals use to enhance their mental health in relation to their job (e.g., setting work–life boundaries, adapting the workspace to one’s needs, and setting small and realistic objectives [21,22]. Individuals with mental health concerns who use work-related MHS strategies have reported reduced psychiatric symptoms, interference in their daily lives due to their mental health symptoms, workplace functioning challenges, and work-related distress, as well as increased energy, self-efficacy, self-esteem, health behaviors, well-being at work, and overall health [43,44]. Furthermore, workers who use these practices find it easier to work than when they are not using the strategies [45]. Finally, workers who use these practices (vs. those who do not) are less likely to quit or take time off work [45].

Previous research has shown that individuals who have an increased awareness of their workplace needs, including mental health ones, are more likely to act on fulfilling them [46]. They are also more likely to utilize self-management techniques, such as seeking help and support from colleagues, informing their manager about their difficulties, and adjusting their work environment to meet their needs [46]. Finally, supportive workplaces facilitate the use of self-management strategies; however, many employers lack the knowledge and the resources to support their employees with chronic long-term mental health disorders. This is partly due to a lack of information as well as mental health stigma, the lack of workers’ disclosure of mental health-related issues they are facing, and adverse job demands [47].

So far, research on mental health self-management at work has focused on the use of these strategies by individuals diagnosed with and/or recovering from mental health-related concerns. However, as suggested by research in the field of self-care, most of these strategies could be used by the general working population (i.e., those without a diagnosed mental health condition) to maintain and improve their well-being despite experiencing negative PWFs. Self-care, defined as “the process of actively initiating a method to promote holistic well-being” [48] (p. 189), shares many similarities with mental health self-management but is broader in scope. While some authors consider self-management a subset of self-care strategies focused on managing an existing illness [34], others appear to use them interchangeably and/or without any clear-cut definitions [49,50]. In addition, although some strategies are explicitly related to mental health disorder management and relapse prevention (e.g., taking one’s medication as prescribed, consulting mental health professionals), other strategies, such as managing stress and maintaining work–life boundaries, can be helpful regardless of their specific/initial intent (i.e., holistic well-being or illness management/relapse prevention), making the distinction unclear. In this paper, we have chosen to employ the concept of mental health self-management strategies, as our focus is on mental health.

Most research on work-related self-care practices used by individuals without a diagnosed mental health condition involves medical and mental health professionals and students (e.g., in the medicine, psychology, social work fields). This line of research has demonstrated that self-care can help prevent burnout and promote overall well-being [48,51,52,53]. Additionally, self-care has been found to moderate the relationship between perceived stress and job burnout among nurses [54]. However, very little is known about the use of self-care practices in other occupations [51] or about the use of MHSW strategies in the general population. Two pan-Canadian surveys aimed at studying the mental health of workers and managers in small and medium-sized enterprises showed that most participants employed MHSW strategies [55,56]. However, these studies did not examine the role of these strategies in moderating the relationship between PWFs and well-being.

### 1.2. Research Gap and Contributions

In sum, some evidence suggests that there are potential benefits to using self-care and/or mental health self-management strategies to help buffer the effects of stress on well-being and prevent negative and/or promote positive mental health outcomes [54]; however, research on this topic involving the general population is sparse. In addition, to our knowledge, no research has explicitly studied MHSW’s role in moderating the relationship between PWFs and negative and positive mental health outcomes.

The present study contributes to the self-management at work literature by showing how MHSW moderates the relationship between certain PWFs and mental health outcomes. It also provides new empirical data by examining these associations in a large Canadian sample comprising diverse workers across various industries and occupations. The study also contributes to the broader field of mental health at work, adopting an approach inspired by Keyes’ [57] mental health continuum model. It examines both negative and positive well-being outcomes, as well as perceived performance, in relation to PWFs and the use of self-management strategies. Finally, the study proposes a more holistic view of adults’ mental health by incorporating both work-related (e.g., positive well-being at work, burnout) and general (e.g., flourishing, anxiety) indicators of well-being.

### 1.3. Objectives and Hypotheses

The present study has two primary objectives:

1. To investigate the relationship between psychosocial work factors and both positive and negative well-being indicators.
2. To explore the association between self-management at work and well-being, as well as the moderating role of self-management at work in mitigating the deleterious effects of psychosocial work factors on well-being.

Concerning these objectives, we posit two accompanying hypotheses. Regarding objective #1, we hypothesized that greater psychological demands and less competency-related autonomy, decisional authority, coworker and supervisor support, and recognition would be associated with worse well-being (H1). Regarding objective #2, we hypothesized that a higher level of self-management at work would be related to better well-being scores (H2). While some research has explored self-care’s moderating role in supporting worker well-being [54], no hypotheses regarding self-management at work’s moderating role(s) were formed as there is a lack of research exploring its role in buffering workplace psychosocial work factors’ effects on well-being.

## 2. Methods

### 2.1. Research Design

The main study from which the current research stems was longitudinal, occurring at two time points during the COVID-19 pandemic: October and November 2020 (T1) and July and August 2021 (T2). The current study employs a cross-sectional design, utilizing data collected at T2 to investigate correlations between variables related to our research objectives.

### 2.2. Participants

Eligible participants were those at least 18 years old, able to read and understand French, and working at least 20 h per week before the onset of the COVID-19 pandemic or at the time of the survey. The participants were required to work at least 20 h per week to ensure that they were sufficiently exposed to the PWFs being measured. The participants were recruited at Time 1 via a hybrid of quota sampling (electronic invitations to Léger Marketing’s LEO online panel) and convenience sampling (emails to Laval University staff). When recruiting LEO participants, an effort was made to achieve representativeness in terms of age, gender, administrative region, and racial and immigration status. The same participants were invited to participate in the T2 survey. The Time 2 responses were used for the analyses. In total, we received 1059 T2 responses. Of these 1059 participants, however, 163 were removed for failing one or more attention checks. Thus, our final sample contained 896 Francophone workers.

### 2.3. Measures

The survey included several self-reported measures related to well-being, the workplace, and demographics, all of which the participants answered in French. Whereas most measures were presented to every participant, the self-management at work measure was only presented randomly to half the participants to increase the number of measurement scales that could be included in the larger study, as the other scales were also presented alternatively. For each measure below, we report the Cronbach’s alphas for the (sub)scales with five or more items and the average inter-item correlation (IIC) for the subscales with two items, as a lower number of items can negatively affect Cronbach’s alpha [58]. For any scales with three or four items, given that a low number of items can still negatively affect the scales’ reliability (e.g., see [59]), we report the average IIC when the Cronbach’s alpha is unacceptable. Relevant to mean MIICs, items will ideally correlate between 0.20 and 0.40, as this suggests adequate internal consistency [60,61]. However, an MIIC above 0.40 is also acceptable. As the subscales in Biron et al.’s [62] workplace psychosocial work factor measure have a small number of items per construct, we justify our decisions regarding item retention when statistics suggest incongruent results.

Psychosocial Work Factors. Biron et al.’s [62] workplace psychosocial work factor measure, based on measures used in the Quebec Survey on Working Conditions, Employment, and Occupational Health and Safety [11], was employed to investigate various psychosocial work factors. In total, 19 of the 23 items found within the scale were adopted to explore six different psychosocial work factors. Items across all the psychosocial work factor subscales were measured on a five-point Likert scale from 1 (Strongly Disagree) to 4 (Strongly Agree). Psychological demands were measured using three items (e.g., “I am asked to do too much work” [9]. The subscale had a non-acceptable internal reliability (α = 0.53) and an acceptable MIIC (*r* = 0.27). While building the measurement model, however, two of the three items (psychosocial work factors 7 and 8) were non-significantly loading onto the psychological demands latent construct (*p*s = 0.14 and 0.13). Due to this, psychological demands were represented in the model as the single exemplary item above as an observable item. This item, from a face validity perspective, seemed to be an adequate single item to capture part of the psychological demands (i.e., workload) construct. Competency-related autonomy (i.e., the opportunity to be creative and to use and develop skills at work [11]), conceptualized as one of the two sub-dimensions of decision latitude, was measured using three items (e.g., “My work requires me to learn new skills” [9]). The subscale had a non-acceptable internal reliability (α = 0.58) and an acceptable MIIC (*r* = 0.32). Given that the items significantly loaded onto the competency-related autonomy latent construct and had an acceptable MIIC, all three items were retained. Decisional authority was measured using two items (e.g., “In my job, I have the freedom to decide how I do my work” [63]). The subscale had a less ideal, but still acceptable, MIIC (*r* = 0.49). Coworkers’ support was measured using three items (e.g., “At my workplace, I feel like I belong to a team” [64]. The subscale had acceptable internal reliability (α = 0.61) and an acceptable MIIC (r = 0.34). Supervisors’ support was measured using three items (e.g., “My supervisor pays attention to what I am saying” [63]). The subscale had good internal reliability (α = 0.75). Recognition was measured using five items (e.g., “Given all my efforts and accomplishments, I get the respect and esteem I deserve at work” [63]). The subscale had good internal reliability (α = 0.74).

Self-management at Work. Self-management at work was explored using 10 items developed by the research team. The developed questions reflect 10 self-management strategies identified in a qualitative study by Roberge et al. [22], which workers used to promote their functioning at work. The items (e.g., “Maintaining a work–life balance”, “Expressing my needs and expectations more assertively”) were answered on a five-point Likert scale ranging from 1 (Never) to 5 (All of the time). The scale’s internal reliability was good (α = 0.83). The measure was randomly administered to half of the participants to increase the number of measures that could be included in the larger study on which this article is based.

Well-being. Six well-being constructs were examined to provide a more holistic understanding of workers’ well-being. Well-being at work, burnout, and work performance measures were used to gain a deeper understanding of workers’ workplace well-being. Anxiety, depression, and flourishing measures were employed to better comprehend workers’ overall well-being in their daily lives.

Positive Well-being at Work. Gilbert and Malo’s [65] nine-item French version of Gilbert et al.’s [66] Psychological Health at Work Scale was used to measure positive well-being at work. The items (e.g., “These days, in my job… ‘I want to do lots of things’, ‘I feel appreciated by others’”) were measured on a seven-point scale from 1 (Never) to 7 (Always). The scale’s internal reliability was excellent (α = 0.94).

Burnout. Sassi and Neveu’s [67] French version of Shirom and Melamed’s [68] Shirom–Melamed Burnout Measure was used to investigate worker burnout. The scale contains fourteen items, of which we used nine. The nine items were grouped by burnout dimension: fatigue (physical; e.g., “I feel physically drained”), cognitive (e.g., “I feel I’m not thinking clearly”), and emotional (e.g., “I feel I am not capable of investing emotionally in coworkers and customers”). These items were measured on a seven-point Likert scale ranging from 1 (Never) to 7 (Always). The overall scale’s internal reliability was excellent (α = 0.94).

Work Performance. Given its good validity in previous studies [69], a single item from URC Eco Ile de France’s translation [70] of the French Health and Work Performance Questionnaire (HPQ [71,72]) was used to measure workers’ job performance. The single item (“Using the same 0-to-10 scale, how would you rate your overall job performance on the days you worked during the past 4 weeks [28 days]?”) was answered on an 11-point Likert scale ranging from 0 (Worst performance) to 10 (Top performance).

Anxiety and Depression. Anxious and depressive symptoms were measured using the French Patient Health Questionnaire-4 (PHQ-4 [73]). The PHQ-4 contains four items: two measuring anxiety (e.g., “Feeling nervous, anxious or on edge”) and two measuring depression (e.g., “Feeling down, depressed or hopeless”). These items were measured on a four-point Likert scale ranging from 1 (Never) to 4 (Nearly Every Day), with higher scores indicating experiencing more anxiety and/or depression. The overall scale’s internal reliability was good (α = 0.89), and the anxiety and depression subscale MIICs were acceptable (*r*s = 0.75 and 0.79).

Flourishing. Villieux et al.’s [74] French version of Diener et al.’s [75] Flourishing Scale was adopted to measure workers’ sense of flourishing in life. The scale contains eight items (e.g., “I lead a purposeful and meaningful life”, “I am optimistic about my future”) answered on a seven-point Likert scale ranging from 1 (Strongly Disagree) to 7 (Strongly Agree). Higher scores indicate more flourishing. The scale’s internal reliability was excellent (α = 0.91).

### 2.4. Procedure

This study was approved by Wilfrid Laurier University’s (REB #6497, 18-03-2020) and Laval University’s research ethics boards (#2020-248, 21-08-2020). Interested workers at T1 first consented to the study and answered questions to ensure their eligibility. By consenting to participate in the survey, the workers agreed to be re-contacted for the Time 2 survey. The consenting and eligible participants then answered several measures regarding sociodemographics (e.g., age, gender), well-being (e.g., sense of flourishing, distress, positive well-being at work), work (e.g., psychosocial work factors, job precarity), and COVID-19 (e.g., infection history). Participants were emailed (Laval University staff) or internally provided with (LEO panel participants) a survey link in July and August 2021 to participate in the T2 survey (i.e., the survey whose data we use for this article). Except for the sociodemographic measures, the participants answered the same questions as those in T1. The LEO participants were compensated at each time point with pre-determined rewards (e.g., gift cards, PayPal transfers) set by Léger Marketing; Laval University staff were entered into a draw for a $75 electronic gift card for every 50 participants.

### 2.5. Data Analysis

The data preparation and descriptive and correlational analyses were completed using SPSS (version 28.0) [76]. The study’s primary analyses were conducted using the MPlus software (version 8.6) [77]. The data analysis plan closely follows the procedure undertaken by Coulombe et al. [78]. Structural equation modelling (SEM) was conducted to address the study’s objectives. The constructs measured by more than one observable variable (i.e., measured items) were included in the SEM as latent constructs [79]. The observable variables were loaded onto their respective latent construct, except psychological demands and work performance, which were measured using single items. As these constructs were directly observable, they were added to the model as such (i.e., they were not included as a latent variable). Except for the moderation analyses, four fit indices were investigated to determine the fit of our SEMs: the Comparative Fit Index (CFI ≥ 0.90), Tucker Lewis Index (TLI ≥ 0.90), Root Mean Square Error of Approximation (RMSEA ≤ 0.07), and Standardized Root Mean Square Residual (SRMR ≤ 0.08) [80]. In addition to checking these fit indices, modification indices (MIs) were also requested and investigated while building the measurement SEM to determine if adding correlational pathways between the scale items would improve the model fit. Such correlational pathways were only included if they made theoretical sense. In total, six correlational pathways between the items were included: (i) “My colleagues have a hostile or conflictual attitude towards me” and “My supervisor has a hostile or conflictual attitude towards me” (MI: 145.57), (ii) “Setting boundaries regarding the time and energy I can dedicate to work” and “Maintaining a work–life balance” (MI: 36.30), (iii) “Reflecting on my environment and working conditions to identify sources of stress” and “Expressing my needs and expectations more assertively” (MI: 33.70), (iv) “I feel appreciated by others” and “I enjoy my relationships” (MI: 38.88), (v) “I am in good spirits” and “I feel emotionally balanced” (MI: 69.71), and (vi) “I feel appreciated by others” and “People respect me” (MI: 58.28). In addition, the modeling included (1) the Robust Maximum Likelihood (MLR) estimator to handle non-normality present within the data [79], (2) the Full-Information Maximum Likelihood (FIML) approach that is part of the MPlus software (v. 8.6) to deal with missing values [81], and (3) the ITERATIONS function, which was set to 10,000.

Addressing research objective #1 and part of objective #2, after the measurement model was built containing all the (in)dependent variables, the main SEM model was built by loading each dependent construct onto each psychosocial work factor and self-management at work. This allowed us to see the direct relationships between our main variables. Only the significant and trending relationships are physically depicted in figures. Further addressing research objective #2, the interactions between the psychosocial work factors and self-management at work were tested across six models. Each model contained (1) all the pathways—significant or not—between the psychosocial work factors, self-management at work, and well-being outcomes, and (2) the interaction term between a psychosocial work factor and self-management at work loaded onto each well-being outcome. For each significant or trending interaction, simple slopes [82] were added to the Mplus syntax to better understand the relationships between the psychosocial work factors and well-being at low, moderate, and high levels of self-management at work.

## 3. Results

### 3.1. Preliminary Analyses and Descriptive Statistics

Although we provide the descriptive statistics for both the full sample and the participants who only completed the self-management at work question, we describe the demographic breakdown solely for the full sample for brevity. The workers had a mean age of 43.20 years. Most workers identified themselves as women, White, Canadian, residing in Quebec, and as having no disabilities, and were either employed or self-employed full-time. Moreover, a larger proportion of participants indicated being in a common-law relationship and working in the professional, scientific, and technical services industry compared to other categories. See Table 1 for the sample’s full demographic breakdown.

Irrespective of the used measures’ conceptual importance and uniqueness, analyses were also conducted to establish their statistical validity. Convergent validity was assessed using computed average variance extracted (AVE) scores for each latent construct [83,84]; discriminant validity was assessed by (i) comparing the latent constructs’ squared-rooted AVE with the average correlation the latent construct had with other latent and observable constructs [85], and (ii) conducting a heterotrait–monotrait (HTMT) ratio analysis [86]. Except for the AVE scores for competency-related autonomy (0.33), coworkers’ support (0.36), recognition (0.41), and self-management at work (0.30), the remaining latent constructs’ AVE scores (0.50–0.87)—including burnout’s first- and second-order latent constructs—were acceptable (≥0.50 [84]). Regarding discriminant validity, the square-rooted AVEs surpassed the average correlations each of the latent constructs had with the other latent and observed constructs combined. The computed HTMT ratio (0.48) was also lower than recommendations (<0.85 [86]), indicating acceptable discriminant validity. Thus, whereas some latent constructs had suboptimal convergent validity, they all displayed acceptable discriminant validity. Although displaying suboptimal convergent validity, all the latent constructs were retained given their importance in the previous literature and their sufficient discriminant validity (see Section 4.2 for more information regarding the limitations associated with construct measurement). Using Harman’s single-factor test [87], confirmatory factor analysis was conducted to assess potential common method bias across the adopted measures. The results of this test indicated that the variance explained (*R*^2^) across the items (30.47%) did not surpass the 50% threshold [87], indicating that a serious threat to the validity of our results was unlikely.

Table 2 contains the descriptive and correlational statistics for the main study variables. Based on an absolute value of 1 on the skewness and kurtosis statistics [88] and a QQ plot inspection, most variables were normally distributed. This was except for self-management at work being kurtotic, as well as supervisors’ support, anxiety, depression, and work performance, all of which were skewed and kurtotic. Although non-normality is present in the study’s variables, the MLR estimator used during model building is robust to non-normality [79]. Most had between 0.00% and 26.10% missing data on the main study constructs, except for self-management at work, which had 55.40% missing data. The responses from about half of the participants had missing data on the self-management at work measure because it was randomly displayed to half of the study’s participants. Although approximately half of the participants had missing data on the self-management at work measure, as described in the “Participants” subsection of the Methods section, the full sample (*N* = 896) was used to address all the study’s objectives more effectively.

### 3.2. Main Analyses

Addressing the two research objectives, a model was tested where the individual pathways between the psychosocial work factors (psychological demands, competency-related autonomy, decisional authority, coworkers’ and supervisors’ support, recognition) and self-management at work were loaded onto each well-being indicator (positive well-being at work, burnout, anxiety, depression, flourishing, work performance). Shown by the fit indices, the model fits well (χ^2^(1510) = 3504.71, TLI = 0.91; CFI = 0.92; RMSEA = 0.04 (90% CI [0.04, 0.04]); SRMR = 0.06).

**Figure 1:**
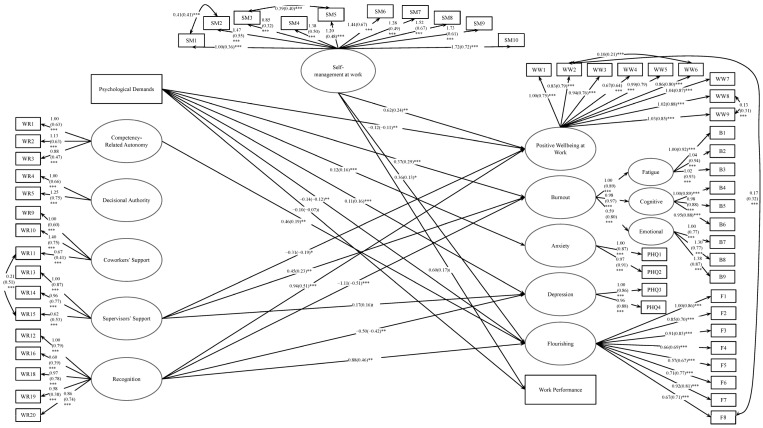
Final Direct Pathways Between Psychosocial Work Factors and Well-being Indicators. Note. Based on *R*^2^ values, 45.30% of positive well-being at work, 31.40% of burnout, 7.90% of anxiety, 13.20% of depression, 31.50% of flourishing, and 19.00% of work performance are explained in this model. *** *p* ≤ 0.001, ** *p* ≤ 0.01, * *p* ≤ 0.05, ^t^ *p* ≤ 0.10.

The significant relationships between these constructs are presented in Figure 1. Providing some support for H1, psychological demands were negatively related to positive well-being at work, flourishing, and work performance; however, the relationship with work performance was trending (i.e., marginally significant, *p* < 0.10). Psychological demands were also positively associated with burnout, anxiety, and depression. Competency-related autonomy was positively related to flourishing.

Recognition was positively related to positive well-being at work and flourishing and negatively related to burnout and depression. Unexpectedly, supervisors’ support was negatively associated with positive well-being at work and positively related to burnout and depression. Decisional authority and coworkers’ support were unrelated to the well-being indicators. Providing some support for H2, self-management at work was positively related to positive well-being at work, flourishing, and work performance.

#### Moderation Analyses

Six moderation analyses were conducted to address the second research objective. Each model tested whether the interaction between a different psychosocial work factor and self-management at work would be related to the six well-being indicators. As shown in Table 3, each interaction between the different psychosocial work factors and self-management at work was associated with well-being at work. Except for the interactions between psychological demands and self-management, which had a positive relationship with positive well-being at work, the other interactions between psychosocial work factors and self-management at work had a negative relationship with positive well-being at work. The interaction between psychological demands and self-management at work was also significantly negatively related to anxiety and depression, as well as being significantly positively associated with work performance. Although not significant, the interactions of supervisor support and recognition with self-management at work had a trending positive relationship with depression. Similarly, the interactions of competency-related autonomy and decisional authority with self-management at work had a trending negative relationship with flourishing. Organized by dependent variable below, the simple slopes for these significant and trending relationships are examined to gain a deeper understanding of these interactions.

Positive Well-being at Work. The relationship between psychological demands and positive well-being at work, based on a simple slope analysis, is negative and the worst at low levels of self-management at work (*b* = −0.48; SE = 0.13; *p* < 0.001 (95% CI [−0.74, −0.22])). At moderate levels of self-management, a negative relationship exists between demands and work well-being. Still, this relationship is smaller than at low self-management levels (*b* = −0.13; SE = 0.04; *p* = 0.002 (95% CI [−0.22, −0.05])). At high self-management levels, demands are associated with higher well-being at work levels (*b* = 0.22, SE = 0.11, *p* = 0.05 (95% CI [0.00, 0.43])).

The relationship between competency-related autonomy and positive well-being at work is positive at low self-management at work levels (*b* = 0.97, SE = 0.44, *p* = 0.03 (95% CI [0.10, 1.84])). There is no association between competency-based autonomy and positive well-being at work at moderate (*b* = 0.28, SE = 0.17, *p* = 0.11 (95% CI [−0.06, 0.61])) and high levels (*b* = −0.42, SE = 0.28, *p* = 0.13 (95% CI [−0.96, 0.12])) of self-management at work.

The relationship between decisional authority and positive well-being at work is positive at low self-management at work levels (*b* = 1.08, SE = 0.38, *p* = 0.004 (95% CI [0.34, 1.82])) and negative at high self-management of work levels (*b* = −0.72, SE = 0.30, *p* = 0.02 (95% CI [−1.31, −0.13])). There is no relationship between decisional authority and positive well-being at work at moderate self-management at work levels (*b* = 0.18, SE = 0.16, *p* = 0.26 (95% CI [−0.13, 0.50])).

The relationship between coworkers’ support and positive well-being at work is positive at low self-management at work levels (*b* = 1.30, SE = 0.64, *p* = 0.04 (95% CI [0.05, 2.55])). There is no relationship between coworkers’ support and positive well-being at work at moderate (*b* = 0.42, SE = 0.42, *p* = 0.31 (95% CI [−0.39, 1.24])) and high (*b* = −0.45, SE = 0.37, *p* = 0.22 (95% CI [−1.17, 0.27])) self-management at work levels.

There is no relationship between supervisors’ support and positive well-being at work at low self-management at work levels (*b* = 0.24, SE = 0.24, *p* = 0.32 (95% CI [−0.23, 0.70])). There is a trending negative relationship between supervisors’ support and positive well-being at work at moderate self-management at work levels (*b* = −0.27, SE = 0.15, *p* = 0.08 (95% CI [−0.57, 0.03])). There is a stronger negative relationship between supervisors’ support and positive well-being at work at high self-management at work levels (*b* = −0.78, SE = 0.19, *p* < 0.001 (95% CI [−1.16, −0.41])).

The association between recognition and positive well-being at work is positive and is the best at low self-management at work levels (*b* = 1.49, SE = 0.34, *p* < 0.001 (95% CI [0.82, 2.16])). The relationship between recognition and positive well-being at work is positive but weaker at moderate (vs. low) self-management at work levels (*b* = 0.94, SE = 0.29, *p* = 0.001 (95% CI [0.37, 1.51])). There is no relationship between recognition and positive well-being at work at high self-management levels (*b* = 0.38, SE = 0.32, *p* = 0.23 (95% CI [−0.24, 1.01])).

Anxiety. The association between psychological demands and anxiety is positive and is the worst at low self-management at work levels (*b* = 0.32, SE = 0.11, *p* = 0.003 (95% CI [0.11, 0.54])). There is a positive relationship between psychological demands and anxiety at moderate self-management at work levels (*b* = 0.12, SE = 0.04, *p* < 0.001 (95% CI [0.06, 0.19])). Still, it is smaller than at low self-management levels. There is no relationship between psychological demands and anxiety at high self-management at work levels (*b* = −0.07, SE = 0.09, *p* = 0.43 (95% CI [−0.26, 0.11])).

Depression. The relationship between psychological demands and depression is positive and is the worst at low self-management at work levels (*b* = 0.36, SE = 0.12, *p* = 0.002 (95% CI [0.13, 0.58])). There is a positive relationship between psychological demands and depression at moderate self-management at work levels (*b* = 0.12, SE = 0.03, *p* < 0.001 (95% CI [0.06, 0.18]), but it is smaller than at low self-management levels. There is no relationship between psychological demands and depression at high self-management at work levels (*b* = −0.12, SE = 0.10, *p* = 0.21 (95% CI [−0.32, 0.07])).

The interaction between supervisors’ support and self-management at work on depression was trending. There was no relationship between supervisors’ support and depression at low (*b* = −0.16, SE = 0.23, *p* = 0.49 (95% CI [−0.60, 0.29]) and moderate (*b* = 0.15, SE = 0.11, *p* = 0.18 (95% CI [−0.07, 0.37]) self-management at work levels. Supervisors’ support was positively related to depression at high self-management at work levels (*b* = 0.46, SE = 0.18, *p* = 0.01 (95% CI [0.10, 0.82])).

The interaction between recognition and self-management at work on depression was trending. The relationship between recognition and depression is negative and was the best at low self-management at work levels (*b* = −0.81, SE = 0.28, *p* = 0.004 (95% CI [−1.35, −0.27])). The relationship between recognition and depression was weaker but still negative at moderate self-management levels (*b* = −0.50, SE = 0.20, *p* = 0.01 (95% CI [−0.90, −0.10])). There was no relationship between recognition and depression at high self-management levels (*b* = −0.20, SE = 0.27, *p* = 0.46 (95% CI [−0.72, 0.32])).

Flourishing. The interaction between competency-related autonomy and self-management at work on flourishing was trending. The relationship between competency-based autonomy and flourishing is positive and is the best at low self-management at work levels (*b* = 1.16, SE = 0.49, *p* = 0.02 (95% CI [0.21, 2.12])). The positive relationship between competency-based autonomy and flourishing is smaller at moderate (vs. low) self-management at work levels (*b* = 0.54, SE = 0.20, *p* = 0.01 (95% CI [0.15, 0.93])). There is no relationship between competency-related autonomy and flourishing at high self-management at work levels (*b* = −0.08, SE = 0.34, *p* = 0.82 (95% CI [−0.74, 0.58])).

The interaction between decisional authority and self-management at work on flourishing was trending. There is no relationship between decisional authority and flourishing at low (*b* = 0.56, SE = 0.38, *p* = 0.15 (95% CI [−0.20, 1.31])) nor moderate (*b* = −0.01, SE = 0.18, *p* = 0.95 (95% CI [−0.36, 0.34])) self-management at work levels. The relationship between decisional authority and flourishing is negative but trending at high self-management at work levels (*b* = −0.58, SE = 0.31, *p* = 0.06 (95% CI [−1.19, 0.03])).

The interaction between recognition and self-management at work on flourishing was trending. The relationship between recognition and flourishing is positive and is the best at low self-management at work levels (*b* = 1.18, SE = 0.35, *p* = 0.001 (95% CI [0.50, 1.86])). The positive relationship between recognition and flourishing is smaller at moderate (vs. low) self-management at work levels (*b* = 0.88, SE = 0.31, *p* = 0.01 (95% CI [0.27, 1.50])). There is no relationship between recognition and flourishing at high self-management at work levels (*b* = 0.58, SE = 0.38, *p* = 0.12 (95% CI [−0.16, 1.32])).

Work Performance. The relationship between psychological demands and work performance is negative and is the worst at low self-management at work levels (*b* = −0.66, SE = 0.22, *p* = 0.003 (95% CI [−1.09, −0.23])). There is a non-significant, but trending, negative relationship between psychological demands and work performance at moderate self-management at work levels (*b* = −0.11, SE = 0.06, *p* = 0.08 (95% CI [−0.23, 0.01]), but it is smaller than at low self-management levels. There is a positive relationship between psychological demands and work performance at high self-management at work levels (*b* = 0.44, SE = 0.18, *p* = 0.01 (95% CI [0.09, 0.80])).

## 4. Discussion

This study aimed to investigate the relationship between psychosocial work factors and both positive and negative well-being at work and in general. We also explored mental health self-management at work’s (1) relationship with positive and negative well-being and (2) its moderating role in preventing negative psychosocial workplace factors’ deleterious effects on well-being. In line with these aims, we tested two hypotheses: (1) that more psychological demands and less competency-related autonomy, decisional authority, coworkers’ and supervisors’ support, and recognition would be related to worse well-being (H1), and (2) that a higher level of self-management at work would be related to better well-being scores (H2). In contrast to our predictions, only some support was found for both hypotheses. Regarding H1, only some PWFs (psychological demands, competency-related autonomy, supervisors’ support, recognition) were related to workers’ well-being, of which supervisors’ support was not in the expected direction. Regarding H2, self-management at work was only positively related to positive well-being at work, flourishing, and work performance. In line with previous research [7], our results suggest that some PWFs (i.e., psychological demands, competency-related autonomy, supervisor’s support, recognition) are related to well-being. Of these, psychological demands was the only factor related to each of the explored well-being outcomes.

Of the well-being indicators explored, self-management was only related to positive ones and not to negative ones. These results contrast with those obtained in research on populations with diagnosed mental health conditions such as depression, for whom mental health self-management strategies appear to include the reduction in depressive symptoms [43].

In addition, self-management did not buffer the relationship between PWFs and burnout. These findings align with previous research suggesting that these strategies may help buffer the effects of stress, but only to a certain point, i.e., as an early preventive measure to preserve well-being [53]. Thus, self-management should not be viewed as a preventive measure against adverse mental health outcomes, such as burnout [89].

This study also found that self-management at work significantly moderated the relationship between each psychosocial work factor and well-being at work. In particular, self-management can play both a beneficial or a potentially harmful role in the relationship between PWFs and well-being outcomes. Indeed, self-management beneficially moderates the relationship between psychological demands and positive well-being at work, anxiety, depression and performance. Interestingly, psychological demands are related to more positive mental health at work where there are high levels of self-management. This suggests that individuals with higher job control and autonomy may have more opportunities to utilize SM strategies, which is generally consistent with the JDR model [7]. Further studies could test this hypothesis.

On the other hand, the use of self-management strategies was found to play a maladaptive role in the relationship between recognition and positive well-being at work, flourishing, and depression. It appears to play a similar role in the relationship between coworkers’ support and competency-related autonomy and positive well-being at work, as well as in the relationship between decisional authority and positive well-being at work and flourishing. Finally, supervisor support was associated with higher levels of depression at high self-management levels. This could indicate that depressed individuals receive more support, a hypothesis that could also be tested in future studies. Alternatively, previous research suggests that employees can experience adverse well-being outcomes despite receiving supervisor support when they perceive the supervisor as being the source of the stressors (e.g., assigning a high workload [90]) or when supervisor support (e.g., helping get the job done) is paired with undermining behavior (e.g., micromanaging, criticizing [91]). In these types of situations, perhaps workers tend to use self-management strategies more often to cope with undesired supervisor behaviors. Further studies could include more comprehensive measures of supervisor support and management practices to assess how each of these can influence the use of self-management strategies and employee well-being outcomes.

Finally, psychological demands were also negatively associated with work performance at low self-management levels but positively associated with performance at high self-management levels. Although this could suggest that self-management has an important role in this relationship, it could also indicate that individuals with higher job control simply have more resources available to them (e.g., autonomy) to engage in work-related self-management strategies, such as “maintaining a work–life balance” [92], which is consistent with the JDR model [7]. Further research could investigate the mechanisms underlying this relationship; for example, do high demands lead to the need for high self-management (SM) for workers to maintain higher performance levels? Is high SM made possible by high job control?

In summary, in line with previous research on self-care and burnout, self-management at work may be more effective in fostering positive well-being at work than in protecting against adverse mental health outcomes, such as burnout [53,89]. The only exception was that self-management at work was shown to significantly moderate the relationship between psychological demands and depression. This may be due to the use of strategies such as establishing work–life balance and taking control over one’s workspace, which could be related to job control and individual regulatory strategies shown to help buffer the effects of high psychological demands, as shown in previous research using the JDR model [7]. Further research could explore this relationship to establish a clearer distinction between the use of self-management strategies as a function of job control and self-management in and of itself.

Overall, MHSM appears to be beneficial for individuals in the workplace because it can help them cope with stressors [48]. In using individual strategies, workers demonstrate and reinforce self-efficacy, a concept that has been at the core of self-management since its inception [24]. Self-efficacy means that a person believes they can control various aspects of a situation to change it [24,93]. As such, self-management practices may enhance an individual’s real or perceived control over their situation and promote resilience [54]. Higher self-efficacy, in turn, has been linked to improved health status [24].

This study contributes to the field of occupational health psychology by examining the potential benefits of using mental health self-management at work for the general working population. Indeed, previous studies have either focused on mental health self-management in individuals with diagnosed mental health conditions or on self-care used by health and mental health professionals. In addition, it provides empirical quantitative data on this subject, which is rare [22].

### 4.1. Practical Implications

In addition to other forms of supervisor support, managers should adopt attitudes and behaviors that support workers’ use of self-management strategies, such as displaying empathy, promoting honest communication, and showing a willingness to find solutions adapted to individual workers’ needs [21,50]. Furthermore, managers and other leaders, including senior management, must lead by example by clearly communicating their commitment to workplace well-being, aligning organizational policies with promoting workplace mental health, and openly adopting practices that promote their own well-being, such as work–life balance, stress reduction activities, and refraining from sending late-night emails [94,95]. Leaders can thus help create a workplace environment that actively promotes the disclosure and self-management of mental health-related issues, as disclosure can provide certain benefits related to self-management strategies, such as obtaining social support and workplace accommodations [96]. However, individuals are less likely to disclose at work when the supervisory style is focused on sanctions and reprimands [97]. These risks may be reduced by providing training and workshops for managers and employees aimed at increasing mental health literacy and reducing stigma [98].

All this being said, just like self-care and stress-management techniques, using self-management strategies should be viewed as complementary to—and not a replacement for—improving PWFs at their source [21,89,94,99]. For example, given that maintaining work–life balance is generally regarded by workers as being one of the most useful self-management/strategies [21] and that it is positively associated with mental health more generally [100], organizations should develop and enforce policies and practices that facilitate involvement in different life spheres throughout workers’ careers [92]. Finally, given how psychological demands were shown to be the only factor related to each of the well-being outcomes explored in this study, targeting this PWF, in particular, should be a priority for organizations wishing to improve employee well-being. A vast body of research, for example, within the JDR tradition, provides a substantial list of specific practices organizations can adopt and of their positive individual and organizational outcomes [7].

### 4.2. Limitations

This study has some limitations that must be acknowledged. First, the sample consisted of individuals who worked at least 20 h per week before and during the COVID-19 pandemic, and thus it excludes individuals for whom self-management strategies may not have been used or were not successful in maintaining a high enough level of mental health to remain at work. It also excludes individuals who stopped working during the pandemic, such as those who lost their jobs due to factors related to this event. Also related to the sample, a mix of quota and convenience sampling was used to recruit participants. The convenience sampling could have biased these results. Secondly, although we used a measure of psychosocial work factors that was part of the Quebec Survey on Working Conditions, Employment, and Occupational Health and Safety [62], some of the internal reliabilities were suboptimal. Some measures also had suboptimal convergence validity (competency-related autonomy, coworkers’ support, recognition, self-management at work), whereas other constructs (psychological demands, work performance) were measured using a single item. With these measurement limitations in mind, future research should aim to replicate these findings with more reliable measures. Furthermore, this research is cross-sectional, making it impossible to establish causality. Additionally, our analyses did not control for occupations which can influence PWFs and workers’ ability to use self-management strategies, depending on individual and workplace characteristics. Finally, having been conducted during the COVID-19 pandemic, the study’s results may not be fully generalizable to non-crisis contexts.

### 4.3. Future Research Directions

Avenues for further research include using larger, more representative samples, including workers who are currently not working due to mental health concerns (e.g., burnout) and comparing individuals with and without formal diagnoses (e.g., clinical level anxiety, depression). In addition, occupational groups could be compared to determine whether certain job characteristics, such as job control, can play a role in the relationship between PWFs, self-management strategies, and well-being outcomes. Further research should also explore how modifying workplace characteristics (e.g., PWFs, psychosocial safety climate, management styles) can facilitate or hinder the use and efficacy of self-management strategies by both workers and managers.

## 5. Conclusions

We have shown that self-management at work helps promote positive mental health in general and at work despite being exposed to negative PWFs. However, self-management strategies do not effectively moderate the relationship between negative PWFs and negative mental health outcomes, further supporting previous research (e.g., [12,101,102]) indicating that interventions that target workplace factors and job characteristics remain fundamental in fostering mental health at work and preventing negative outcomes such as burnout. This study contributes to the mental health self-management literature by demonstrating how all workers, not just those with clinical diagnoses, can benefit from employing self-management strategies at work to achieve and maintain overall well-being, both personally and professionally. Indeed, although research on mental health self-management and self-care generally focuses on preventing negative mental health outcomes (e.g., burnout), our study contributes to this field by answering Wise’s [103] call for the integration of a positive dimension, allowing workers to flourish rather than merely survive. We also contribute to a more holistic picture of working adults’ lives by exploring their well-being in both their work and personal lives. Finally, public health organizations and employers should continue to promote mental health literacy in general, while also providing education and training to foster the development and use of self-management strategies, particularly in the workplace.

## Figures and Tables

**Table 1:** Sample Characteristics.

Variables	Frequency (%) or Mean (SD)	
	Participants Who Answered SM Questions (*n* = 400)	Full Sample (*N* = 896)
AGE (YEARS)	43.75 (11.03)	43.20 (11.92)
GENDER		
Men	165 (41.30)	336 (37.50)
Women	234 (58.50)	556 (62.10)
Non-binary	0 (0.00)	1 (0.10)
Missing/Prefer not to say (%)	1 (0.30)	3 (0.30)
MARITAL STATUS		
Married	112 (28.00)	250 (27.90)
Common law	151 (37.80)	336 (37.50)
Never married	108 (27.00)	239 (26.70)
Separated	6 (1.50)	17 (1.90)
Divorced	20 (5.00)	39 (4.40)
Widowed	1 (0.30)	12 (1.30)
Other (e.g., Fiancé, Couple living separately)	2 (0.50)	3 (0.30)
IDENTIFIED AS A PERSON OF COLOR		
Yes	16 (4.00)	38 (4.20)
No	382 (95.50)	854 (95.30)
Prefer not to say (%)	2 (0.50)	4 (0.40)
BORN IN CANADA		
Yes	364 (91.00)	816 (91.10)
No	36 (9.00)	80 (8.90)
PROVINCE OF RESIDENCE ^a^		
Alberta	1 (0.30)	1 (0.10)
New Brunswick	0 (0.00)	1 (0.10)
Ontario	3 (0.80)	5 (0.60)
Prince Edward Island	1 (0.30)	1 (0.10)
Quebec	395 (98.80)	887 (99.00)
Saskatchewan	0 (0.00)	1 (0.10)
HAS AT LEAST ONE DISABILITY		
Yes	20 (5.00)	55 (6.10)
No	380 (95.00)	837 (93.40)
Missing/Prefer not to say (%)	0 (0.00)	4 (0.40)
EMPLOYMENT STATUS ^a^		
Employed/self-employed full-time (30+ hours/week)	326 (81.50)	693 (77.30)
Employee or self-employed (20–29 h/week)	34 (8.50)	83 (9.30)
Employee or self-employed (<20 h/week)	27 (6.80)	70 (7.80)
On leave for physical or mental health problems	9 (2.30)	2 3(2.60)
Retired	1 (0.30)	6 (0.70)
Full-time student	8 (2.00)	21 (2.30)
Part-time student	15 (3.80)	20 (2.20)
Unemployed and looking for work	5 (1.30)	18 (2.00)
Unemployed and not looking for work	2 (0.50)	6 (0.70)
Receiving employment insurance benefits	4 (1.00)	12 (1.30)
Receiving disability benefits	2 (0.50)	4 (0.40)
Social assistance recipient	0 (0.00)	1 (0.10)
Other (e.g., Maternity leave, COVID-19-related layoff)	2(0.50)	11(1.20)
WORK INDUSTRY ^a^		
Retail	33 (8.30)	67 (7.50)
Other services excluding public administration (e.g., repair, maintenance)	23 (5.80)	57 (6.40)
Professional, scientific, and technical services	85 (21.30)	188 (21.00)
Arts, entertainment, and recreation	22 (5.50)	30 (3.30)
Finance and insurance	27 (6.80)	58 (6.50)
Health care and social assistance	49 (12.30)	129 (14.40)
Construction	12 (3.00)	25 (2.80)
Accommodation and food services	5 (1.30)	9 (1.00)
Education	41 (10.30)	81 (9.00)
Wholesale trade	3 (0.80)	15 (1.70)
Manufacturing	19 (4.80)	35 (3.90)
Information and cultural industries	6 (1.50)	14 (1.60)
Transportation and warehousing	5 (1.30)	11 (1.20)
Agriculture, forestry, fishing, and hunting	8 (2.00)	20 (2.20)
Company/Enterprise management	5 (1.30)	7 (0.80)
Real estate and rental and leasing services	4 (1.00)	9 (1.00)
Administrative and support, waste management, and remediation services	14 (3.50)	28 (3.10)
Public administration	44 (11.00)	84 (9.40)
Mining and oil and gas extraction	1 (0.30)	3 (0.30)
Utilities (electricity, gas, and water services)	6 (1.50)	12 (1.30)
Other	1 (0.30)	9 (0.90)
Missing (%)	20 (5.00)	71 (7.90)

Notes. Sample characteristics are based on data collected at T1. ^a^ Participants were able to choose one or more categories. SM = self-management at work.

**Table 2:** Descriptive and Correlational Statistics.

Construct	*N*	*M*	*SD*	*S*	*K*	1.	2.	3.	4.	5.	6.	7.	8.	9.	10.	11.	12.	13.
1. Psychological demands	743	2.28	0.90	0.35	−0.59	-												
2. Competency-related autonomy	792	2.90	0.64	−0.58	0.33	0.07 *	-											
3. Decisional authority	795	3.17	0.69	−0.73	0.32	−0.08 *	0.29 ***	-										
4. Coworkers’ support	690	3.32	0.54	−0.72	0.27	−0.13 ***	0.27 ***	0.31 ***	-									
5. Supervisors’ support	662	3.32	0.64	−1.20	1.56	−0.21 ***	0.20 ***	0.35 ***	0.56 ***	-								
6. Recognition	757	3.00	0.54	−0.39	0.06	−0.26 ***	0.20 ***	0.37 ***	0.56 ***	0.59 ***	-							
7. Self-management at work	400	3.30	0.67	−0.39	1.07	−0.10 ^t^	0.15 **	0.21 ***	0.30 ***	0.27 ***	0.31 ***	-						
8. Positive well-being at work	805	5.29	1.04	−0.68	0.83	−0.24 ***	0.23 ***	0.34 ***	0.43 ***	0.36 ***	0.50 ***	0.38 ***	-					
9. Burnout	803	2.98	1.20	0.26	−0.38	0.38 ***	−0.06	−0.21 ***	−0.30 ***	−0.26 ***	−0.38 ***	−0.16 **	−0.66 ***	-				
10. Anxiety	896	1.31	1.61	1.33	1.20	0.19 ***	−0.01	−0.07 *	−0.10 **	−0.09 *	−0.19 ***	−0.10 ^t^	−0.45 ***	0.55 ***	-			
11. Depression	896	1.17	1.51	1.53	2.02	0.20 ***	−0.05	−0.09 **	−0.15 ***	−0.13 ***	−0.23 ***	−0.15 **	−0.57 ***	0.63 ***	0.71 ***	-		
12. Flourishing	896	5.58	0.94	−0.60	0.21	−0.20 ***	0.23 ***	0.26 ***	0.35 ***	0.32 ***	0.46 ***	0.28 ***	0.71 ***	−0.52 ***	−0.42 ***	−0.55 ***	-	
13. Work performance	801	7.92	1.44	−1.01	1.97	−0.16 ***	0.09 **	0.23 ***	0.29 ***	0.28 ***	0.35 ***	0.24 ***	0.52 ***	−0.45 ***	−0.27 ***	−0.37 ***	0.46 ***	-

Note. *S* = Skewness, *K* = Kurtosis. *** *p* ≤ 0.001, ** *p* ≤ 0.01, * *p* ≤ 0.05, ^t^ *p* ≤ 0.10.

**Table 3:** Test of the Moderating Role of Self-management at Work between Psychosocial Work Factors and Well-being.

	DV: Positive Well-Being at Work				DV: Burnout				DV: Anxiety				DV: Depression				DV: Flourishing				DV: Work Performance			
	*B*	*β*	95% *CI* *[LL*, *UL]*	*D* *R* ^2^	*B*	*β*	95% *CI* *[LL*, *UL]*	*D* *R* ^2^	*B*	*β*	95% CI *[LL*, *UL]*	*D* *R* ^2^	*B*	*β*	95% CI *[LL*, *UL]*	*D* *R* ^2^	*B*	*β*	95% CI *[LL*, *UL]*	*D* *R* ^2^	*B*	*β*	95% CI *[LL*, *UL]*	*D* *R* ^2^
IV: Psychological demands	−0.13 **	−0.12	−0.22, −0.05		0.38 ***	0.29	0.27, 0.48		0.12 ***	0.16	0.06, 0.19		0.12 ***	0.17	0.06, 0.18		−0.15 **	−0.13	−0.24, −0.05		−0.11 ^t^	−0.08	−0.23, 0.01	
MOD: SM at work	0.68 ***	0.25	0.27, 0.34		−0.21	−0.07	−0.63, 0.22		−0.21	−0.11	−0.46, 0.05		−0.17	−0.10	−0.41, 0.07		0.38 *	0.14	0.03, 0.73		0.70 *	0.20	0.14, 1.26	
Interaction term	0.35 **	0.13	0.12, 0.57	0.016	0.02	0.01	−0.34, 0.38	0.007	−0.20 *	−0.11	−0.39, −0.01	0.015	−0.24 *	−0.14	−0.44, −0.04	0.020	0.18	0.07	−0.06, 0.42	0.003	0.55 **	0.16	0.18, 0.93	0.024
IV: Competency-related autonomy	0.28	0.12	−0.06, 0.61		0.07	0.03	−0.30, 0.44		−0.03	−0.02	−0.29, 0.22		−0.08	−0.06	−0.33, 0.16		0.54 **	0.23	0.15, 0.93		0.07	0.02	−0.40, 0.53	
MOD: SM at work	0.62 **	0.23	0.19, 1.04		−0.23	−0.07	−0.64, 0.19		−0.17	−0.09	−0.42, 0.08		−0.05	−0.03	−0.28, 0.19		0.43 *	0.15	0.05, 0.80		0.57 ^t^	0.16	−0.02, 1.16	
Interaction term	−0.70 *	−0.12	−1.34, −0.05	0.010	−0.58	−0.09	−1.76, 0.60	0.018	−0.02	−0.01	−0.49, 0.45	0.003	0.33	0.09	−0.27, 0.93	0.002	−0.62 ^t^	−0.11	−1.34, 0.10	0.016	−0.80	−0.11	−2.10, 0.49	0.001
IV: Decisional authority	0.18	0.08	−0.13, 0.50		−0.14	−0.06	−0.53, 0.25		−0.04	−0.03	−0.33, 0.25		0.03	0.02	−0.23, 0.29		−0.01	−0.01	−0.36, 0.34		0.43 ^t^	0.15	−0.07, 0.94	
MOD: SM at work	0.65 ***	0.25	0.27, 1.04		−0.19	−0.06	−0.59, 0.21		−0.16	−0.09	−0.40, 0.08		−0.09	−0.05	−0.31, 0.14		0.42 **	0.15	0.09, 0.75		0.64 *	0.18	0.04, 1.25	
Interaction term	−0.90 **	−0.17	−1.49, −0.31	0.016	−0.18	−0.03	−1.36, 1.01	0.009	0.26	0.07	−0.28, 0.80	0.001	0.45	0.13	−0.26, 1.15	0.001	−0.57^t^	−0.11	−1.15, 0.02	0.000	−0.96	−0.14	−2.27, 0.35	0.015
IV: Coworkers’ support	0.42	0.16	−0.39, 1.24		−0.11	−0.04	−1.03, 0.80		−0.03	−0.02	−0.66, 0.60		−0.04	−0.03	−0.66, 0.57		0.04	0.01	−0.76, 0.83		0.81	0.24	−0.35, 1.97	
MOD: SM at work	0.75 ***	0.27	0.30, 1.21		−0.22	−0.07	−0.66, 0.23		−0.18	−0.09	−0.45, 0.09		−0.15	−0.08	−0.41, 0.11		0.47 *	0.16	0.09, 0.84		0.73 *	0.19	0.07, 1.39	
Interaction term	−0.88 **	−0.13	−1.49, −0.27	0.018	−0.41	−0.05	−1.62, 0.80	0.015	0.33	0.07	−0.26, 0.91	0.000	0.45	0.10	−0.24, 1.14	0.001	−0.54	−0.08	−1.23, 0.16	0.001	−0.57	−0.06	−1.71, 0.56	0.012
IV: Supervisors’ support	−0.27 ^t^	−0.16	−0.57, 0.03		0.41 *	0.21	0.05, 0.77		0.05	0.04	−0.19, 0.29		0.15	0.14	−0.07, 0.37		−0.09	−0.05	−0.38, 0.20		−0.09	−0.04	−0.51, 0.32	
MOD: SM at work	0.69 **	0.25	0.25, 1.12		−0.19	−0.06	−0.62, 0.24		−0.17	−0.09	−0.42, 0.09		−0.10	−0.05	−0.34, 0.14		0.43 *	0.15	0.06, 0.80		0.65 *	0.18	0.03, 1.27	
Interaction term	−0.51 ***	−0.12	−0.81, −0.21	−0.001	−0.13	−0.03	−0.86, 0.59	0.006	0.24	0.08	−0.06, 0.54	−0.001	0.31 ^t^	0.11	−0.03, 0.64	−0.005	−0.26	−0.06	−0.64, 0.12	−0.003	−0.30	−0.05	−0.87, 0.28	−0.003
IV: Recognition	0.94 ***	0.52	0.37, 1.51		−1.15 ***	−0.53	−1.81, −0.49		−0.35	−0.28	−0.81, 0.11		−0.50 **	−0.43	−0.90, −0.10		0.88 **	0.47	0.27, 1.50		0.47	0.19	−0.24, 1.17	
MOD: SM at work	0.74 ***	0.28	0.32, 1.16		−0.23	−0.07	−0.66, 0.20		−0.20	−0.10	−0.45, 0.06		−0.16	−0.09	−0.41, 0.08		0.46 **	0.17	0.10, 0.82		0.70 *	0.20	0.09, 1.30	
Interaction term	−0.55 ***	−0.12	−0.86, −0.24	0.020	−0.18	−0.03	−0.88, 0.51	0.016	0.26	0.08	−0.05, 0.56	0.008	0.31 ^t^	0.11	−0.05, 0.66	0.009	−0.30 ^t^	−0.06	−0.66, 0.06	0.005	−0.39	−0.07	−0.97, 0.19	0.005

Note. *B* = Unstandardized beta, *β* = Standardized beta, *CI* = Confidence interval, *LL* = Lower limit, *UL* = Upper limit, *DR*^2^ = R^2^ change in dependent variable, SM = self-management. Confidence intervals regarding the unstandardized beta estimates are provided. R^2^ change is calculated as the difference in the variance explained when compared to the same dependent variable in the SEM model in Figure 1 (i.e., a model where the individual interactions are not included). *** *p* ≤ 0.001, ** *p* ≤ 0.01, * *p* ≤ 0.05, ^t^ *p* ≤ 0.10.

## Data Availability

The data presented in this study are available on request from the corresponding author.
